# Abdominal Challenging Image in an Elderly Woman

**DOI:** 10.4274/balkanmedj.2016.1364

**Published:** 2017-08-04

**Authors:** Vitorino Modesto dos Santos, Filipe Emanuel Fonseca Menezes

**Affiliations:** 1 Catholic University Medical Course, Brasilia-DF, Brazil; 2 Armed Forces Hospital, Brasilia-DF, Brazil

A 72-year-old Brazilian woman with hypertension, diabetes, dyslipidaemia, hyperuricaemia, hypothyroidism, obesity, and alcohol abuse had dyspnoea, cough, and vomiting for 3 weeks. Drugs in use: amlodipine, indapamide, atenolol, losartan, simvastatin, metformin, glimepiride, allopurinol and levothyroxine. No previous abdominal invasive studies or surgical procedures. Physical examination was unremarkable. Laboratory: normal haemoglobin, haematocrit, and platelets; leukocytes 12.940/mm^3^, eosinophils 776/mm^3^; normal proteins, transaminases, calcium, thyroid-stimulating hormone, free thyroxine 4, lipidogram, and amylase; lipase 111 IU/L; glycated haemoglobin 11.1%, creatinine 2.1 mg/dL, urea 129.3 mg/dL, and C-reactive protein (CRP) 4.7 mg/L. Abdominal X-ray showed a tortuous tubular radiopaque image fixed on the left-upper quadrant (Figure 1a); computed tomography revealed isolated calcification of the splenic artery (Figure 1b). Written informed consent was obtained from the patient.

Main differential diagnoses include a foreign body - the lack of history of invasive procedures (Gastroenteral tubes and ventriculoperitoneal shunt) discarded this hypothesis and an ascaris lumbricoides, which causes vomiting, recurrent dyspnoea, dry cough (Löffler’s syndrome). She lived in rural areas where ascaridiasis is prevalent; but the images were outside of the intestine. Calcifications of chronic pancreatitis - she was an alcohol abuser, but the calcification was restricted to the splenic artery (Figure 1b). As the lipase level was elevated, with normal amylase, she had probably suffered a recent mild asymptomatic episode of recurrent pancreatitis. 

The observed tortuosity was due to a growing difference between the arterial length and the distance from its origin and the splenic hilum (1). Calcification of this artery is often similar to the peripheral arteries of diabetic or dialytic patients (1); however, her lipid profile was normal and no calcific plaques were seen in the aorta and main branches, as described (2). Media calcification involves concentric diffuse mineral deposition sparing the lumen, and intima calcification of atherosclerotic plaque causes ischaemia by arterial narrowing (3). Tortuous calcified splenic artery is not rare in the elderly, with calcification in the media (1). Atherosclerotic plaque is the most frequent type in the splenic artery (4). Similar plaques in the abdominal aorta coexist with splenic artery calcifications in up to 89.5% of individuals; furthermore, the splenic artery presented with a small number of calcified plaques (4).

A recent Turkish study focused on biomarkers of atherosclerosis in young people with a family history of coronaropathy, including CRP and interleukin-6 (5). It was worth noting that carotid intima-media thickness was not useful to evaluate subclinical atherosclerosis among the Turkish adolescent group (5). The patient herein reported kidney dysfunction and high CRP, often related to secondary hyperparathyroidism with bone mobilisation of calcium and multiple arterial calcifications. This mechanism was discarded by normal calcium and isolated calcification of the splenic artery. She successfully used anti-hypertensive and lipid lowering drugs, but glycaemic control did not yield favourable results. Isolated calcification incidentally observed in the splenic artery occurred by unclear mechanisms. This may be exceedingly rare or under-diagnosed. As a similar description was not found in the literature, this clinical image might stimulate reports of diagnosed cases that have not yet been published. 

## Figures and Tables

**Figure 1:**
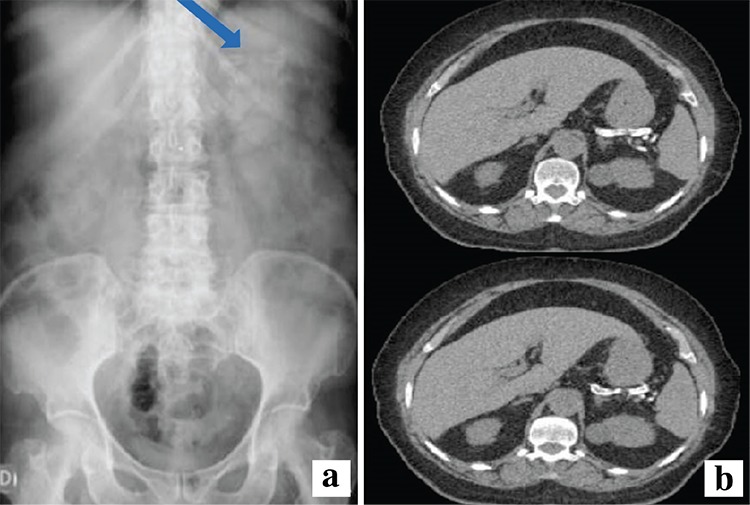
Abdominal plain radiography showing radiopaque tortuous parallel lines with a tubular aspect on the left upper quadrant (a). Computed tomography images confirming the isolated calcified splenic artery (b).
